# The Microbiota-Gut-Brain Axis During Heat Stress in Chickens: A Review

**DOI:** 10.3389/fphys.2021.752265

**Published:** 2021-10-20

**Authors:** Chang Cao, Vishwajit S. Chowdhury, Mark A. Cline, Elizabeth R. Gilbert

**Affiliations:** ^1^Department of Animal and Poultry Sciences, Virginia Polytechnic Institute and State University, Blacksburg, VA, United States; ^2^Laboratory of Stress Physiology and Metabolism, Faculty of Arts and Science, Kyushu University, Fukuoka, Japan

**Keywords:** heat stress, microbiota-gut-brain axis, anorexia, immune response, poultry

## Abstract

Heat stress is a global issue for the poultry industries with substantial annual economic losses and threats to bird health and welfare. When chickens are exposed to high ambient temperatures, like other species they undergo multiple physiological alterations, including behavioral changes, such as cessation of feeding, initiation of a stress signaling cascade, and intestinal immune, and inflammatory responses. The brain and gut are connected and participate in bidirectional communication via the nervous and humoral systems, this network collectively known as the gut-brain axis. Moreover, heat stress not only induces hyperthermia and oxidative stress at the gut epithelium, leading to impaired permeability and then susceptibility to infection and inflammation, but also alters the composition and abundance of the microbiome. The gut microflora, primarily via bacterially derived metabolites and hormones and neurotransmitters, also communicate via similar pathways to regulate host metabolic homeostasis, health, and behavior. Thus, it stands to reason that reshaping the composition of the gut microbiota will impact intestinal health and modulate host brain circuits via multiple reinforcing and complementary mechanisms. In this review, we describe the structure and function of the microbiota-gut-brain axis, with an emphasis on physiological changes that occur in heat-stressed poultry.

## Introduction

The microbiota-gut-brain axis (MGBA) has been widely investigated in human and mammalian species for decades due to its vital role in not only homeostatic maintenance but also the pathology of various neurodevelopmental and neurodegenerative disorders (Cryan et al., 2019). In addition, the importance of this axis in non-mammalian species, such as chickens, has been acknowledged and the potential mechanisms are being investigated. The relationship between the gut microbiome and host is considered to be mutualistic rather than commensal (De Palma et al., 2014). The host provides the microbiome with hospitable niches and undigested food, and in turn, these microorganisms metabolize and produce neuroactive components. These neuroactive molecules, such as serotonin (5-HT), exert a systematic or local effect in regulating host physiological processes, by entering the circulation or interacting with enteric nervous and immune systems, respectively. Factors that act on the central nervous system (CNS), for instance via vagal afferents, influence host behaviors, whereas others trigger structural and functional changes in the intestine (Villageliũ and Lyte, 2017). The interactive effects between the gastrointestinal microbiota and the host could be benign or detrimental, depending on the type and magnitudes of factors, including but not limited to dietary composition, environmental stimuli, and host genetics and phenotypes (Kers et al., 2018).

Stress, by a simple definition, is the adaptive physiological and psychological response of an organism to restore homeostasis (Glaser and Kiecolt-Glaser, 2005). The intestinal tract is reported to be involved in responses to all kinds of stressors, including heat stress (Rostagno, 2020). Heat stress is a major environmental challenge and occurs when there is an imbalance in the net amount of heat energy produced by and released from an organism (Renaudeau et al., 2012), during which the organism accumulates more heat than it can utilize and release. Heat stress is closely related to changes in the intestine, both structurally and functionally, and in the composition of the gut microbiota (Song et al., 2014; Sohail et al., 2015). Studies on the effects of heat stress on the gut microbiota in humans are lacking (Karl et al., 2018), and a variety of animal models is utilized to thoroughly investigate these effects to provide clinical insights. In this review, we focus on avian models, because heat stress has been widely reported to influence poultry meat and egg production, as well as flock health and wellbeing, through major changes in intestinal physiology and the gut microbiota (Lara and Rostagno, 2013; Rostagno, 2020). However, how heat stress interacts with the chicken’s gut microbiota and affects the MGBA is not fully understood and requires further elucidation.

Herein, we review literature related to heat stress-induced alteration in chicken behaviors (such as feeding and social behaviors), physiological processes, intestinal integrity and the microbiota, and the immune system, with an emphasis on the relationships of these alterations to gut microbiota composition. We also review what is known regarding the use of probiotics and prebiotics as preventative and therapeutic interventions in heat-stressed animals, and discuss strategies to ameliorate the detrimental effects of high temperatures on bird behavior and health.

## Microbiota-Gut-Brain Axis

### Microbiome Composition

Gut microbes consist of different microorganisms, such as bacteria, viruses, yeast, and other fungi, and protozoa. Most research on gut microbiota has focused on evaluating bacteria composition and function (Karl et al., 2018); hence, bacteria being the target of this review. The amount of microbiota varies dramatically between intestinal sites, from about 10^5^ colony-forming units (CFU) per gram of digesta in the small intestine to around 10^11^CFU per gram of digesta in the cecum (Xing et al., 2019; Rychlik, 2020). During the past decade, technological advances in profiling microbiomes within the host, from improvements in laboratory culture techniques to 16S rRNA gene sequencing and metagenomics sequencing, have enabled the study of the composition of the microbiome with greater resolution and depth. However, it is important to note that knowing the microbiome composition does not necessarily facilitate an understanding of their function and physiological consequences. In chickens, Lactobacilli dominate several parts of the upper digestive tract, including the crop, proventriculus, and ventriculus (gizzard), whereas the small intestine is mainly inhabited by *Lactobacillus*, *Enterococcus*, and *Clostridiaceae*. This prevalence of specific species is, to some extent, related to the function of the digestive organs, since the pH of gastric juices is relatively low, which favors domination by Lactobacilli. In the cecal tonsils, where digesta resides the longest time during digestion, and the concentration of short-chain fatty acids (SCFAs) synthesized by bacteria is greater than elsewhere in the gastrointestinal tract (GIT), the most abundant phyla are *Firmicutes*, *Bacteroides*, and *Proteobacteria* (Oakley et al., 2014; Villageliũ and Lyte, 2017; Karl et al., 2018; Rychlik, 2020).

### Functions of Microbial Products

Microbial products can serve as an energy source to fuel the host and are capable of interacting with immune or neuroendocrine systems to influence host health and behaviors (Shenderov, 2016). SCFAs, once being taken up, can be used as a metabolic substrate (ATP production) by intestinal cells, particularly intestinal epithelial cells (enterocytes) (Bergman, 1990). Butyrate and propionate, two major SCFAs, interact with specific G-protein-coupled receptors to regulate and maintain energy and immune homeostasis in cells and thus influence their activity, by activating pathways, such as chemotaxis, apoptosis, proliferation, and differentiation, through gene expression programming (Clarke et al., 2014; El Aidy et al., 2016). Acetate and butyrate are reported to participate in the maintenance of GIT barrier intactness, through which bacterial colonization and translocation are prevented (Fukuda et al., 2011; Fachi et al., 2019). Additionally, SCFAs act as signaling molecules and are closely related to the synthesis of a variety of neuroactive molecules, such as leptin, glucagon-like peptide 1, and other hormones, which can be transported through the circulation to several brain regions. Neurons in the arcuate nucleus of hypothalamus, for instance, receive signals through receptors of these neuropeptides and neurotransmitters that are integrated to then regulate the host’s appetite (Tolhurst et al., 2012; Clarke et al., 2014; El Aidy et al., 2016).

Bacteria synthesize classic neurotransmitters, such as 5-HT, which can act locally or distantly through the circulation or nervous system, and as such have been referred to in the literature as “mind-altering” (Cryan and Dinan, 2012). In the intestine, host enterochromaffin cells, a type of entero-endocrine cell, produce 5-HT. While most dietary-derived tryptophan is metabolized in the liver via the kynurenine shunt, some is converted into 5-HT. In fact, the majority (> 95%) of 5-HT in the body is synthesized in the gut, occurring via sequential conversion of tryptophan via two enzymatic reactions. Intestinally derived 5-HT, whether of host or bacterial origin, can then act via the endocrine system or through the vagus nerve. Within the small intestine, most 5-HT is released into the mucosa, and it is estimated that roughly 2% of all enteric neurons are serotonergic (Mawe and Hoffman, 2013). Via a variety of receptors, including the ionotropic 5-HT3 and metabotropic 5-HT1, 2, 4, and 7, 5-HT influences gut motility (peristalsis), secretion of chemicals, such as bicarbonate, during digestion, vasodilation, and neuronal survival and inflammation (Mawe and Hoffman, 2013).

### Relationship Between Microbiota and the Host Gut

Under normal and healthy conditions, microbial communities in the host GIT play an overall beneficial role. They assist in competing against pathogenic microbial taxa and maintaining intact intestinal lumen surface structures, ferment undigested polysaccharides into SCFAs, and provide additional vitamins (Oakley et al., 2014). Indeed, coprophagic species, such as rabbits and rodents, recover such vitamins by consuming fecal pellets. There are detrimental effects of the gut microbiota undergoing dysbiosis, which can be initiated by but also exacerbated in response to gastrointestinal environment perturbations (temperature, pH, nutrient composition, toxins, introduction of microflora, etc.), resulting in several acute or chronic diseases in the host (Karl et al., 2018). There is a clear relationship, for example, between intestinal diseases, such as Crohn’s disease and inflammatory bowel syndrome, and unbalanced SCFA production and 5-HT availability in the gut (Oligschlaeger et al., 2019). Thus, maintaining a healthy gut microbial community and hospitable mucosal environment is of utmost importance to host health and wellbeing. Generally, a microbiota that is diverse in both composition and genetic content or is dominated by beneficial taxa is characterized as being a healthy community (Karl et al., 2018).

### Physiological Connections Between Gut Microbiota and the Host Brain

The gut microbiota and brain have bidirectional connections. On the one hand, gut microbiota themselves are an important source of peripheral neurotransmitters and hormones. These molecules not only modulate gut functions like peristalsis, as described above, but also directly communicate the intestinal state through vagal afferents to the brainstem and higher brain regions. Various stressful stimuli through peripheral and central pathways lead to the activation of the hypothalamic-pituitary-adrenal (HPA) axis, which might further alter gut microbiota composition and activity as well as intestinal epithelial cells’ function. Release of corticotropin-releasing factor (CRF) from the hypothalamus stimulates adrenocorticotropic hormone (ACTH) release from the anterior pituitary into the circulation, which then triggers the release of corticosteroids from the adrenal glands, including cortisol by humans and corticosterone by birds from the adrenal cortex into the circulation. Corticosteroids exert a multitude of effects on the GIT via direct interactions with enteric muscle cells and neurons, bacteria, and intestinal immune cells, leading to the release of cytokines, which via the circulation can act on the brain to affect mood, appetite, cognition, and emotion (Cryan and Dinan, 2012). Several environmental factors, such as dietary composition and drugs, can influence activity of the MGBA through one or more pathways that feed into these mutually reinforcing connections. For instance, appetite regulation is mediated mainly in several brain regions like the hypothalamus and brainstem. Nutrients in the gut stimulate the release of satiety factors, such as cholecystokinin, and also directly affect the microbiota, which in turn regulate the concentration of cytokines and neuroactive molecules that modulate brain function (Petra et al., 2015).

## Heat Stress

The adaptive physiological and behavioral responses of an organism to environmental demands or pressures have been described as stress responses, by which the organism attempts to maintain or restore homeostasis (De Palma et al., 2014; Karl et al., 2018). Stressors or stressful stimuli can vary from acute to chronic and from one time to several repetitive occurrences, and their magnitude can be mild or severe. Additionally, the different capabilities of individuals to perceive stress result in various outcomes (Lucassen et al., 2014). Individuals exposed repeatedly to stressful situations appear to be more vulnerable to gastrointestinal diseases.

There exist various factors that cause changes in the intestinal microbiota of chickens. One major source of these factors is characteristics of the host itself, such as age, type and breed, sex, and sampling site in the GIT. Environmental factors also influence the microbiota composition, including biosecurity level, housing condition, litter, feed, temperature, and location (Kers et al., 2018). Among those environmental factors, a growing amount of evidence indicates that heat stress has significant effects on the intestinal microbiota composition and tissue structure (see these effects via altered concentrations of neural/humoral factors in Table 1).

**Table 1:** The effects of heat stress-induced neuronal/humoral factors on intestinal physiology and gut microbiota.

Factors	Effects	Species	References
HSF, HSP, and TLR	Induced oxidative stress and intestinal barrier breakdown, initiated inflammatory signaling	Chicken	Varasteh et al., 2015
Proinflammatory cytokines (e.g., IL-1β, IL-2, IL-6, and TNF-α)	Damaged tight junction and gut epithelial integrity, activated HPA axis, enhanced successful transmission of pathogens	Chicken	Al-Sadi et al., 2010; Deng et al., 2012; Alhenaky et al., 2017; Jiang et al., 2021
Corticosterone	Altered HPA axis, increased macrophage oxidative burst and decreased numbers of macrophage undergoing phagocytosis, depressed immune response, induced intestinal lesions, altered gut microbial communities	Chicken	Deng et al., 2012; Quinteiro-Filho et al., 2012b; Beckford et al., 2020; Zaytsoff et al., 2020
Monoamines (e.g., 5-HT, NE, E, and DA)	Increased corticosterone and inflammatory cytokines	Chicken, rat	Johnson et al., 2005; Bahry et al., 2017
Appetite-related neuropeptides (e.g., CCK, ghrelin, CRF, and NPY)	Reduced food intake, activated HPA axis and stress response, impaired intestinal structure	Chicken	Lei et al., 2013; Bohler et al., 2020; Wang et al., 2021
Reactive oxygen and/or nitrogen species	Abnormal heat tolerance, injured intestinal barrier, invading pathogens, translocated endotoxins, increased inflammatory cytokines	Rat	Hall et al., 2001

5-HT, 5-hydroxytryptamine; CCK, cholecystokinin; CRF, corticotropin-releasing factor; DA, dopamine; E, epinephrine; HPA axis, hypothalamic-pituitary-adrenal axis; HSF, heat shock factor; HSP, heat shock protein; IL, interleukin; NE, norepinephrine; NPY, neuropeptide Y; TLR, toll-like receptor; and TNF-α, tumor necrosis factor-α.

When birds are exposed to stressful factors (such as long-term exposure to sunlight, high ambient temperature and humidity, and poor ventilation), their internal energy homeostasis is disrupted and physiological alterations ensue. The transient or long-term imbalance between heat dissipation to the environment and heat production inside the animal can disturb thermostasis and eventually result in heat stress. The thermoneutral zone is the ambient temperature range where the animal efficiently regulates and maintains a constant body temperature (Pollock et al., 2021). When environmental temperature exceeds the upper critical temperature, which is the upper limit of the thermoneutral zone, animals are considered to be exposed to heat stress (McNab, 2002). In general, thermoneutral zones for broiler chickens are 28~34, 25~31, 22~28, 20~25, 18~24, and 18~24°C for each of the first six weeks of age, respectively (Cassuce et al., 2013).

Core body temperature, when elevated by exposure to high ambient temperature, surprisingly did not dramatically alter the microbiota in the cecal tonsils (Xing et al., 2019). However, another study (Alhenaky et al., 2017) found that compared with the thermoneutral condition, both acute and chronic heat stress led to a higher rectal temperature, with the magnitude even higher in the former situation. Rectal temperature peaked during the first two days of heat exposure, then fluctuated until it reached a plateau. After that, individuals under heat stress showed thermo homeostasis during the rest of the observation period. The prevalence of intestinal pathogens (*Salmonella* spp.) was increased in heat-exposed chicks in comparison with the control birds (Alhenaky et al., 2017). These results suggest that core body temperature, despite being temporarily affected by heat exposure, could be adjusted promptly and exert a limited direct effect on the gut microbiota. However, the capacity for adaptation might be compromised if the chickens are exposed to consistent high ambient temperatures after the first few days, which can lead to a severe susceptibility to heat stress.

**Figure 1:**
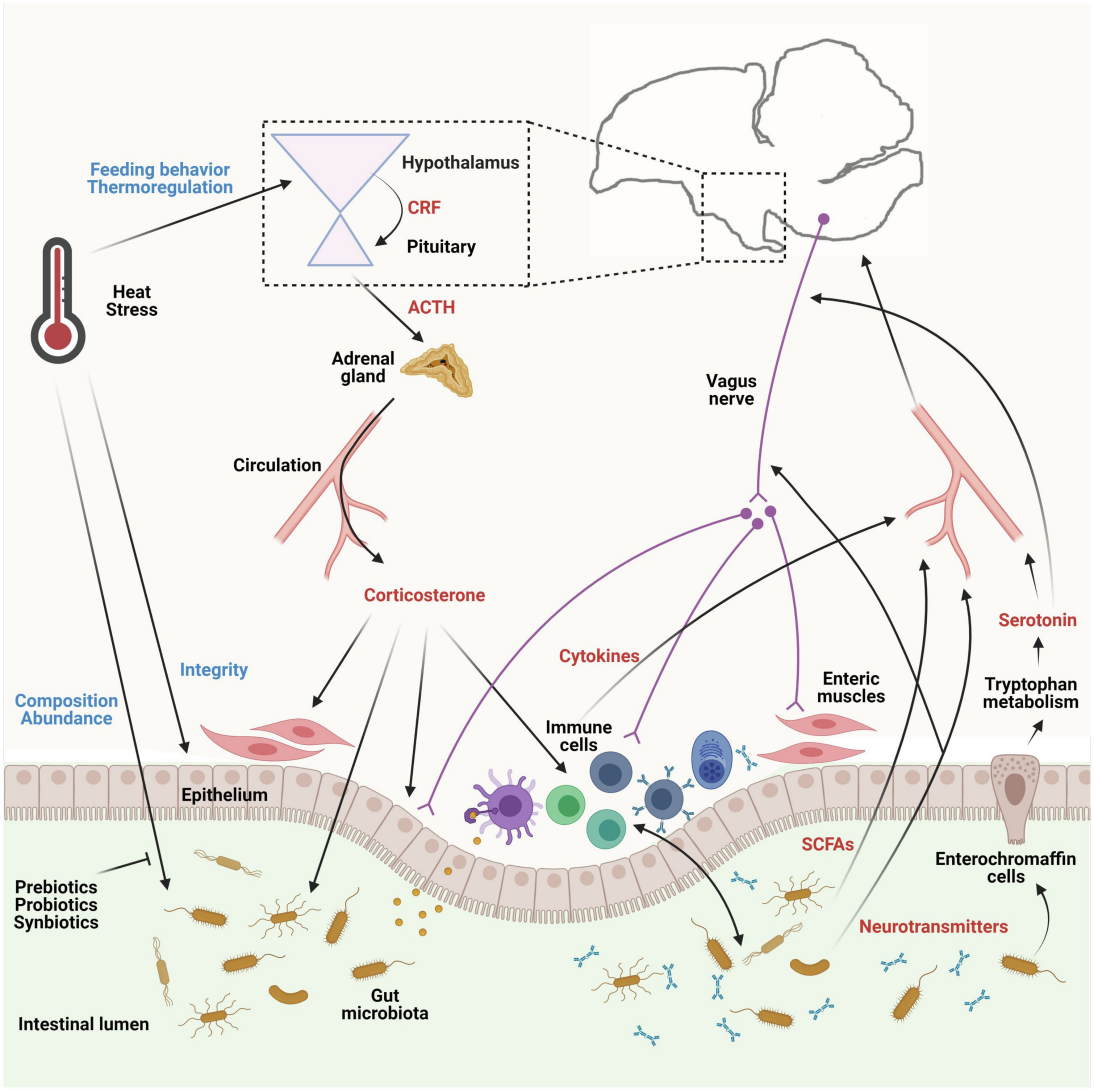
Influences of heat stress on the microbiota-gut-brain axis (MGBA) and the pathways involved in the axis in chicken. The gut microbiota communicates with the gut-brain axis through several pathways, including neural, immune, and endocrine signaling. The gut microbiome produces neurotransmitters, such as serotonin, which can trigger responses of the vagus and enteric nervous system, and short-chain fatty acids (SCFAs), which can nourish the host and regulate the host’s brain activity and behaviors. Gut microbiota also stimulates intestinal immune cells to generate and secrete cytokines to induce immune response locally and systemically and modulate the brain. In turn, the brain utilizes the same pathways to alter the gut microbiota, especially their composition and abundance, under circumstances, such as heat stress. Heat stress can influence the MGBA both directly and indirectly. The hypothalamic-pituitary-adrenal axis is activated by heat stress and facilitates the production of corticosterone in birds, which further affects enteric cells and gut microbiota. The utilizations of prebiotics, probiotics, or symbiotics are promising approaches to alleviate the adverse effects of heat stress. ACTH, adrenocorticotropic hormone; CRF, corticotropin-releasing factor. This figure is adapted from previous publications (Cryan and Dinan, 2012; Aoki et al., 2017) and created with BioRender.com.

Thus, the influence of heat stress on the composition of the gut microbiota can occur as a direct consequence of altering body temperature, or indirectly due to an acute or gradual change in the birds’ behavior, physiological status, intestinal integrity, and immune system activity (See Figure 1), which will be discussed in detail in the following sections.

## Heat Stress Induces Appetite Suppression

Our group demonstrated that exposure to high ambient temperatures suppressed food consumption in young broiler chickens, which was associated with changes in the activity of several appetite-regulating peptides, such as orexigenic neuropeptide Y and anorexigenic CRF, which both have peripheral effects associated with the enteric system and HPA axis (Bohler et al., 2020). In another heat stress study, heat-exposed birds ate less, ingested more water, panted more often, and lifted the wings much longer (Mack et al., 2013). Typically, the reduction in food intake is sustained during heat stress and is thought to be a compensatory mechanism to reduce heat production associated with nutrient metabolism, although heat stress is associated with changes in nutrient absorption, particularly amino acids and glucose. For this reason, a multitude of heat stress studies with chickens and other species have employed dietary strategies to mitigate nutrient-induced heat production, including formulating diets to vary in macronutrient composition (Chowdhury et al., 2021). The GIT of chickens consumes about 7% of the energy derived from the diet, so reduced feeding, while offsetting some of the heat production in the animal, could also elicit a fast and dramatic response in the GIT, primarily jeopardizing gut integrity and mucosal immunity, which further compromises nutrient assimilation, triggers systemic inflammation, and impairs production (Thompson and Applegate, 2006; Deng et al., 2012).

In some commercial practices, the distance of the grow-out facility from the brooder house necessitates transporting chicks over long distances after hatching and processing. Although the residual yolk sac provides a reservoir of nutrients that are resorbed into the intestine and used by the chick after hatching, delayed access to food after hatch can impair intestinal development (Lamot et al., 2014; Proszkowiec-Weglarz et al., 2019, 2020; Qu et al., 2021), and also establishment of the microbiota (Flint et al., 2012). Similar influences were observed in response to food withdrawal at a later age (Burkholder et al., 2008). Having no access to food, even for a period of 6h, allows pathogens, like *Salmonella* (Burkholder et al., 2008), to colonize within the gut and reshape the microbial community (Thompson et al., 2008). Sequencing techniques used to evaluate the taxonomy of the gut microbiota revealed that chickens subjected to food withdrawal had altered populations in the ileum and cecum, with increased *Firmicutes* and diminished *Proteobacteria*. Overall, the major effects of food deficiency on the intestinal microbiome are on the dominant families, such as *Turicibacteraceae*, *Ruminococcaceae*, and *Enterobacteriaceae* (Metzler-Zebeli et al., 2019). In broiler breeders, it is common practice to restrict the amount of food consumed throughout life, in order to meet target body weights to prevent metabolic disorders and support optimal reproduction. However, as described above, such practices could negatively impact the gut microbiome which in turn could impact health of the chicken. Combined with exposure to high temperatures, restricted access to feed could have major impacts on the bacterial composition of the GIT and thereby affect the bird’s whole-body physiology.

Heat stress also negatively affects layer-type chicks, by reducing their food consumption, egg production, and quality, and increasing death rate (Mack et al., 2013; Mignon-Grasteau et al., 2015; Sahin et al., 2018). In laying hens, integrity of the gut mucosa was impaired under heat exposure, resulting in limited nutrient transport across the intestinal mucosal layer (Zhang et al., 2017). Moreover, the intestinal microbiome community was modified in heat-stressed pullets and hens (Burkholder et al., 2008; Song et al., 2014; Zhu et al., 2019). Xing et al. (2019) found that layer chicks displayed an altered microbiome composition rather than species abundance, in response to exposure to a high ambient temperature (29–35°C), and this change was closely associated with less food consumption. Another study involved exposing the layers to a cyclic temperature of 35°C 7h per day and found an increased alpha diversity, that is, the present species of the microbiome were elevated in the cecum after 2weeks of exposure, although they returned back to normal levels after 4weeks (Hsieh et al., 2017). Additionally, the two most abundant cecal phyla, *Bacteroidetes* and *Firmicutes*, showed different richness by the end of the experiment. This study suggested that heat stress started to reshape the microbiota in layers at 2weeks but the bacteria adapted to the temperature change later at 4weeks (Hsieh et al., 2017). Shi et al. (2019) found slightly different results and observed significant changes in the abundance of those two phyla starting at 1week, although also a loss of significance by 4weeks. All of these findings suggest that the influence of heat stress on the gut microbiota depends on the magnitude and duration of heat exposure.

In layer chicks under heat exposure, there were elevated numbers of several detrimental genera, including *Escherichia*, *Shigella*, and *Clostridium*, which generate alpha-toxins and contribute to the occurrence of necrotizing enterocolitis. On the other hand, advantageous bacteria, such as *Lactobacillus* and *Ruminococcaceae*, were scarce (Heida et al., 2016). Bacteria in the genus *Lactobacillus* are widely used as probiotics, as their metabolites are capable of regulating the acid-base equilibrium in the intestine, which favors the development of a beneficial but not pathogenic microbiome (Menconi et al., 2011). Some species in the *Lachnospiraceae* group are also inhibited during heat stress (Biddle et al., 2013; Meehan and Beiko, 2014). These species produce a relatively large proportion of butyrate, which helps maintain intestinal health by facilitating epithelial development. Generally, butyrate is less abundant than other SCFA (60% acetate, 25% propionate, and 15% butyrate; at least in humans), although it serves as the major energy source for colonocytes in the large intestine and is known to affect gene expression by acting as a histone deacetylase (HDAC) inhibitor and affects signaling via activation of several G-protein-coupled receptors (Liu et al., 2018). Many studies have demonstrated a beneficial role for butyrate in maintaining intestinal barrier integrity, and in preventing inflammation (Liu et al., 2018). Thus, changes in the numbers of butyrate-producing bacteria could modulate the MGBA via effects of butyrate signaling on the peripheral and CNS (Liu et al., 2018).

## Heat Stress Reduces Intestinal Integrity

The gut microbiome environment is normally stable under healthy conditions. The intestine provides niches for bacteria to colonize and thrive, and in turn, commensal bacteria compete with pathogenic bacteria for space and nutrients to survive and produce metabolites that boost host intestinal immunity and suppress the growth of pathogens, which collectively protect the gut epithelium. However, stressful stimuli can concurrently impair intestinal barrier integrity and alter the microbiome (Tannock and Savage, 1974; Söderholm et al., 2002; MacDonald, 2005). Once the mucosal layer is penetrated, intestinal pathogens have access to the host circulation and cause diseases and impair the efficiency of nutrient digestion and assimilation (Sansonetti, 2004; Keita and Söderholm, 2010).

There is evidence that the intestinal mucosa, which is susceptible to heat stress and microbiome change-induced damage and inflammation, can also adapt to maximize nutrient assimilation in some circumstances. Heat-treated chicks had decreased plasma thyroid hormone and increased plasma corticosterone, as well as a damaged mucosal layer in the jejunum, but the ability to transport glucose across the jejunal epithelium was enhanced, which may have compensated for the lack of energy due to reduced food consumption (Garriga et al., 2006). In another study, however, when chicks experienced a higher temperature (35°C, 5 degrees higher), their intestinal structures were significantly damaged, with reduced villus heights and functional absorptive surface areas, and elevated levels of blood endotoxins. These adverse impacts were not overcome by host adaptations alone but required exogenous butyrate supply for alleviation of symptoms, further demonstrating a beneficial role for butyrate in maintaining intestinal structure and function (Abdelqader et al., 2017).

The ileum is a unique intestinal niche because of its proximity to the cecum and receipt of end-products of digestion that are not absorbed in the proximal small intestine. It is home to a larger amount of bacteria, even the pathogenic *Salmonella*, than the proximal small intestine and provides a rich source of nutrient substrate for fermentative activity (Fanelli et al., 1971). The intestine stands as the first line of defense against invading pathogens (Fagarasan, 2006). If, for some reason, the chicken small intestinal epithelium is damaged, *Salmonella* adhere at impaired locations and translocate into the host, causing a systemic infection (Burkholder et al., 2008). This was observed in chickens that underwent 24h of food deficiency or heat stress (McHan et al., 1988; Alhenaky et al., 2017). Treating chicks with high temperature chronically or acutely result in invading *Salmonella*, which are later detected in the liver, spleen, and muscles. The liver and spleen typically handle these exogenous pathogens, which are engulfed by macrophages and transported through the circulation. However, the organs that are primarily targeted by *Salmonella* during a systemic infection have yet to be identified (Chappell et al., 2009).

Two mechanisms were proposed that mediate the effect of heat stress on the intestinal epithelium. The first is that reactive oxygen and/or nitrogen species are produced in response to high environmental temperature and increased oxidative activity, overwhelming the capacity of endogenous antioxidant systems (Hall et al., 2001). When chicks are heat-exposed, the production of these free radical molecules provokes injury to the epithelial cell membranes, resulting in fewer tight junctions (TJ) and less expression of TJ genes. Thus, the intestinal barrier becomes permeable to paracellular entry by bacterial endotoxins. The second mechanism is that heat stress promotes the production of proinflammatory cytokines, which also damage the TJ (Al-Sadi et al., 2010). Among those cytokines, interleukin-2 (IL-2) and tumor necrosis factor-α (TNF-α) are among those whose concentrations in circulation are elevated in heat-stressed chicks. IL-2 is produced by T cells, and once released, it activates other types of cells like macrophages, which secrete proinflammatory cytokines, such as TNF-α, to initiate inflammation (Hoyer et al., 2008). However, the secretion of IL-2 could also be stimulated by endotoxins (Costalonga and Zell, 2007); thus, this mechanism might be a secondary or indirect effect.

## Heat Stress Activates the HPA Axis

The HPA axis is an essential system that integrates and mediates an organism’s response to intrinsic and/or extrinsic stressors (McEwen, 2000). Its activation is characterized by the activation of hypothalamic CRF, the release of ACTH, and the production of corticosterone in rodents and birds (Iyasere et al., 2017). Chronic and acute high ambient temperature exposure activate the HPA axis, which is usually characterized by elevated blood corticosterone in the animal. Elevations in circulating corticosterone are associated with an array of physiological responses, such as suppressed food intake and growth performance, and aberrant immune and inflammatory responses, to name a few (Quinteiro-Filho et al., 2012a; Beckford et al., 2020).

In addition to corticosterone, activation of the HPA axis is accompanied by the generation of many other hormones, neuroactive molecules, and cytokines. These factors are shared by many systems in the body (such as the CNS, endocrine, and immune systems) and directionally mediate systematic interplay through the binding of ligands to their receptors (Kaiser et al., 2009). For example, the CNS regulates immunity primarily through HPA axis activity and sympathetic outflow (Ziegler, 2002). Hormones involved in the regulation are corticosterone from the HPA axis and catecholamines from sympathetic activity. Two major catecholamines, norepinephrine (NE) and epinephrine (E), could further regulate the synthesis of inflammatory cytokines, with reduced levels of proinflammatory IL-12, TNF-α, and interferon γ, and enhanced expression of anti-inflammatory IL-10 and transforming growth factor β (Johnson et al., 2005). In turn, signals from visceral organs or tissues, particularly the GIT, can be picked up by parasympathetic inflow or sent back to the HPA axis (Calefi et al., 2016). Indeed, intestinal inflammation provides feedback to the HPA axis, which in turn regulates immune defense against pathogens (Karrow, 2006).

Although activation of the HPA axis by heat stress is linked to intestinal immunity and inflammation (Lara and Rostagno, 2013; Galley and Bailey, 2014; Scanes, 2016; Calefi et al., 2017), few have gone so far as to investigate actual changes in the gut microflora and brain activity. Generally, beneficial commensal bacteria were less competitive, whereas pathogenic species, such as *Escherichia coli* and *Salmonella*, flourished in heat-stressed animals due to impaired intestinal integrity and function, and increased permeability (Song et al., 2013). In a study with broiler chickens, heat stress and/or intestinal infection with *Clostridium* and *Eimeria* spp. (bacteria and protozoal species, respectively) led to changes in concentrations of monoamines in key brain regions, including a decrease in 5-HT, NE, and E in the hypothalamus, and dopamine in the mid-brain (Calefi et al., 2019). Authors speculated that these data demonstrated activation of the HPA axis via increased release of cytokines from intestinal immune cells, in response to the pathogen challenge. Monoamine concentrations and cytokine production in the small intestine were not investigated in that study. Future research should focus more on the connection between neurobiology and the gut microbiome in models of heat- and pathogen-induced intestinal dysfunction.

## Alleviating the Adverse Effects of Heat Stress

A multitude of strategies have been employed to alleviate heat stress in chickens, from improvements in housing management to nutritional interventions, such as varying macronutrient composition and supplementing prebiotics, probiotics, and their combination known as synbiotics (Lara and Rostagno, 2013).

Probiotics, such as live yeasts and/or *Lactobacillus* and *Bifidobacterium*, are usually the dominant beneficial bacteria in the GIT. Exogenous supplementation contributes to maintenance of a healthy gut via ensuring their continued establishment and proliferation, which in turn affects the HPA axis and chicken behavior or physiology via immunomodulation, metabolic homeostasis, and neuroendocrine loops (Wang et al., 2018). The most effective probiotics are usually commensal bacteria belonging to the host (Dogi and Perdigón, 2006). *Bacillus subtilis*, for example, when supplied in the broiler diet, competed with pathogens (*i.e*., *Eimeria* spp. and *Clostridium perfringens*) for colonizing sites and nutrients, thus protecting the gut from their colonization and invasion (Lee et al., 2015). *B. subtilis* was reported to inhibit bacterial pathogenic reproduction and promotes feed utilization by increasing microbiota diversity and promoting the proliferation of the beneficial *Lactobacillus* (Knap et al., 2011). Additionally, *B. subtilis* can stimulate the secretion of intestinal digestive enzymes to speed up nutrient metabolism when the activities of those enzymes were suppressed by chronic heat stress (Chen et al., 2009). Longer villi and larger surface areas were observed in probiotic-supplemented chickens and protected the bird against heat exposure-induced gut dysfunction (Deng et al., 2012; Song et al., 2014).

Prebiotics are generally defined as food ingredients, usually a saccharide, that are not digested (or absorbed) by the host but benefit the host by encouraging the growth of certain species of bacteria for which they serve as fermentative substrates. Common examples include fructo-oligosaccarides (FOS), mannan-oligosaccharides (MOS), and inulin. Mannan-oligosaccharide is harvested from the yeast cell wall and is one of the most common prebiotics used in the poultry industry. Synbiotics are synergistic combinations of prebiotics and probiotics (Schrezenmeir and de Vrese, 2001). Both prebiotics and probiotics exert beneficial effects on animal health when supplemented into the diet (Sohail et al., 2012; Sugiharto et al., 2017; Awad et al., 2021). However, their combination as synbiotics may lead to synergistic and additive effects. Synbiotics not only favor the colonization and thriving of commensal microorganisms, but also activate signaling in the microbiome-gut-brain axis and microbiome-gut-immune axis to mediate systemic and local functions, which further influences host physiology and behavior (Rooks and Garrett, 2016). In one study, broilers were fed a normal diet or synbiotic-supplied diet and exposed to normal or high temperatures. Synbiotic supplementation not only attenuated heat stress-induced anorexia and body weight loss but was also associated with increased preening and decreased panting and wing lifting (Mohammed et al., 2018). Because of the diverse array of probiotic species and prebiotic saccharides and resulting combinatorial possibilities in a synbiotic, synbiotics can have differing effects on the gut microbiome depending on the composition. For instance, when MOS, but not FOS, was used in a synbiotic mixture, different commensal microorganisms were selectively promoted, and MOS was associated with a binding to and elimination of pathogenic bacteria from the GIT (Spring et al., 2000; Sohail et al., 2012).

## Conclusion and Implications

In summary, heat stress induces various physiological alterations that directly or indirectly regulate the intestinal microbiome community. These alterations induce changes in environmental and nutritional conditions in the gut, leading to a breach in the intestinal epithelium or barrier integrity, inflammatory states, and activation of the HPA axis and autonomic nervous system. Although growing evidence links heat stress to changes in the host brain (e.g., monoamine concentrations) and gut that are influenced by alterations in the intestinal microbiota, there are still many gaps in knowledge. For example, most studies focused on the association between heat stress and the chicken gut microbiota, but few confirmed the exact compositional changes of microbiota under different heat stress conditions (such as acute or chronic, one time, or repetitive) or in response to different probiotic and prebiotic interventions in different types, breeds, and ages of chickens. In addition, various metabolites (e.g., SCFAs) and neuroactive molecules (e.g., 5-HT) produced by gut microbiota under different heat stress conditions also require consideration and further exploration. Future studies should focus on utilizing more combinations of probiotics and prebiotics to improve chicken performance under heat exposure and to determine effects on microbiome composition. While it is clear that heat stress influences host and microbial physiology, it is unclear the extent to which the former is driven by the latter and vice-versa. Thus, elucidating the mechanisms that shape the physiology of the GIT and microbiome and how the host and microbial cells interact to drive physiology and behavior will facilitate holistic strategies to ameliorate the effects of heat stress in animals and humans.

## Author Contributions

CC, MC, and EG conceived the idea for the review. CC drafted the manuscript. VC, MC, and EG edited the manuscript. All authors read and approved the final version.

## Funding

Funding for this work was provided in part by the Virginia Agricultural Experiment Station and the Hatch Program of the National Institute of Food and Agriculture, US Department of Agriculture.

## Conflict of Interest

The authors declare that the research was conducted in the absence of any commercial or financial relationships that could be construed as a potential conflict of interest.

## Publisher’s Note

All claims expressed in this article are solely those of the authors and do not necessarily represent those of their affiliated organizations, or those of the publisher, the editors and the reviewers. Any product that may be evaluated in this article, or claim that may be made by its manufacturer, is not guaranteed or endorsed by the publisher.
